# Supplementary material for: From random walks to thermal rides: universal anomalous transport in soaring flights
Source: J R Soc Interface. 2026 Oct 7;23(243):20260227. doi: 10.1098/rsif.2026.0227 (PMC13641945; doi:10.1098/rsif.2026.0227)
Supplement: Supplementary file 1 [file rsif.2026.0227.zip › rsif.2026.0227/rsif20260227_si_001.pdf]

Electronic Supplementary Material: HMM Python Code

README.md

````markdown
# Flight phase HMM code

This folder contains a cleaned implementation of the Hidden Markov Model used to
segment soaring-flight trajectories into flight phases.

## Files

- `trajectory_features.py`: converts raw GPS tracks into three binary features:
  straightness persistence, positive vertical speed, and curvature-sign
  persistence.
- `hmm_categorical.py`: categorical-emission HMM and Baum-Welch / Viterbi
  routines.
- `default_parameters.py`: fitted parameters used for inference.
- `postprocessing.py`: rolling majority filter for smoothing decoded states.
- `infer_flight_phases.py`: command-line inference script for a single track.
- `train_hmm.py`: optional training script from a folder of tracks.
- `simulation_check.py`: synthetic-data sanity check.

## Input data format

Tracks are expected as `.csv`, `.pkl`, or `.pickle` files with columns:

- `time`: time in seconds;
- `Lat`: latitude in degrees;
- `Long`: longitude in degrees;
- `AltGNSS`: GNSS altitude in meters.

Optional columns are preserved in the inference output.

## Example inference

```bash
python infer_flight_phases.py input_track.pkl output_track.csv --glider-type paraglider
```

Valid `--glider-type` values are `paraglider`, `hang_glider`, and `sailplane`.

The output includes two added columns:

- `regime_raw`: raw Viterbi-decoded state labels;
- `regime`: decoded labels after rolling-majority smoothing.

## State and observation ordering

The observation vector is ordered as:

1. straightness persistence indicator;
2. positive vertical-speed indicator;
3. curvature-sign persistence indicator.

The default emission distributions use the following state order:

1. climb;
2. search;
3. transition.

If a different fitted model is used, update `STATE_NAMES` in
`default_parameters.py` accordingly.
````

trajectory_features.py

```python
"""Feature construction for soaring-flight HMM segmentation."""

from __future__ import annotations

from dataclasses import dataclass

import numpy as np
import pandas as pd


@dataclass
class FeatureConfig:
    """Configuration for trajectory preprocessing and binary features."""

    smoothing_window: int = 6
    persistence_window: int = 30
    beta_persistence: float = 0.9
    straightness_threshold_rad: float = 0.20960958


class TrajectoryFeatureFactory:
    """Convert raw GPS tracks into binary HMM observations.

    Expected input columns are ``time``, ``Lat``, ``Long`` and ``AltGNSS``.
    Output columns are ordered as follows:

    1. straightness persistence indicator;
    2. positive vertical-speed indicator;
    3. curvature-sign persistence indicator.
    """

    def __init__(self, config: FeatureConfig | None = None) -> None:
        self.config = config or FeatureConfig()
        self.last_dataframe: pd.DataFrame | None = None

    def transform(self, raw_data: pd.DataFrame) -> np.ndarray:
        """Return HMM observations with shape (T, 3)."""
        df = self._validate_input(raw_data.copy())
        df = self._add_time_columns(df)
        df = self._add_cartesian_coordinates(df)
        df = self._add_features(df)
        self.last_dataframe = df
        return df[
            [
                "persistence_straight",
                "positive_vertical_speed",
                "persistence_curvature_sign",
            ]
        ].to_numpy(dtype=int)

    def _add_cartesian_coordinates(self, df: pd.DataFrame) -> pd.DataFrame:
        df[["x", "y", "z"]] = self.geodetic_to_enu(
            latitude=df["Lat"].to_numpy(),
            longitude=df["Long"].to_numpy(),
            altitude=df["AltGNSS"].to_numpy(),
        )
        for col in ["x", "y", "z"]:
            df[col] = self.smooth_series(df[col], self.config.smoothing_window)
        return df

    def _add_features(self, df: pd.DataFrame) -> pd.DataFrame:
        signed_curvature = self.signed_turning_angle(
            df["x"].shift(1).to_numpy(),
            df["x"].to_numpy(),
            df["x"].shift(-1).to_numpy(),
            df["y"].shift(1).to_numpy(),
            df["y"].to_numpy(),
            df["y"].shift(-1).to_numpy(),
        )
        df["signed_curvature"] = (
            pd.Series(signed_curvature, index=df.index)
            .rolling(window=6, center=True, min_periods=1)
            .mean()
        )
```

```

        dt = df["time"].diff()
        df["vertical_speed"] = df["z"].diff() / dt
        df["mean_vertical_speed"] = (
            df["vertical_speed"]
            .rolling(window=self.config.persistence_window, center=True, min_periods=1)
            .mean()
        )

        is_straight = (
            df["signed_curvature"].abs() < self.config.straightness_threshold_rad
        ).astype(int)
        df["persistence_straight"] = (
            is_straight.rolling(
                window=self.config.persistence_window, center=True, min_periods=1
            ).mean()
            > self.config.beta_persistence
        ).astype(int)

        df["positive_vertical_speed"] = (df["mean_vertical_speed"] > 0).astype(int)
        df["persistence_curvature_sign"] = self.sliding_window_sign_persistence(
            df["signed_curvature"],
            window_size=self.config.persistence_window,
            beta_persistence=self.config.beta_persistence,
        )

        df[
            ["persistence_straight", "positive_vertical_speed", "persistence_curvature_sign"]
        ] = df[
            ["persistence_straight", "positive_vertical_speed", "persistence_curvature_sign"]
        ].ffill().fillna(0)
        return df

    @staticmethod
    def _validate_input(df: pd.DataFrame) -> pd.DataFrame:
        required = {"time", "Lat", "Long", "AltGNSS"}
        missing = sorted(required.difference(df.columns))
        if missing:
            raise ValueError(f"Missing required columns: {missing}")
        return df

    @staticmethod
    def _add_time_columns(df: pd.DataFrame) -> pd.DataFrame:
        df["time_diff"] = df["time"] - df["time"].min()
        return df

    @staticmethod
    def smooth_series(data: pd.Series, window: int) -> pd.Series:
        std = window / 6
        return data.rolling(window=window, win_type="gaussian", center=True).mean(std=std)

    @staticmethod
    def geodetic_to_enu(latitude: np.ndarray, longitude: np.ndarray, altitude: np.ndarray) -> [illegible]
        """Convert WGS84 geodetic coordinates to a local ENU frame."""
        semi_major_axis = 6378137.0
        flattening = 1 / 298.257223563
        eccentricity_sq = flattening * (2 - flattening)

        phi = np.deg2rad(latitude)
        lamb = np.deg2rad(longitude)
        sin_phi = np.sin(phi)
        cos_phi = np.cos(phi)
        radius = semi_major_axis / np.sqrt(1 - eccentricity_sq * sin_phi**2)

        x = (radius + altitude) * cos_phi * np.cos(lamb)
        y = (radius + altitude) * cos_phi * np.sin(lamb)
        z = (radius * (1 - eccentricity_sq) + altitude) * sin_phi
        ecef = np.stack((x, y, z), axis=-1)
        delta = ecef - ecef[0]

        phi0 = np.deg2rad(latitude[0])
        lamb0 = np.deg2rad(longitude[0])
        sin_phi0, cos_phi0 = np.sin(phi0), np.cos(phi0)
        sin_lamb0, cos_lamb0 = np.sin(lamb0), np.cos(lamb0)
        rotation = np.array(
            [
                [-sin_lamb0, cos_lamb0, 0.0],
```

```
                [-sin_phi0 * cos_lamb0, -sin_phi0 * sin_lamb0, cos_phi0],
                [cos_phi0 * cos_lamb0, cos_phi0 * sin_lamb0, sin_phi0],
            ]
        )
        return delta.dot(rotation.T)

    @staticmethod
    def signed_turning_angle(
        x_prev: np.ndarray,
        x_now: np.ndarray,
        x_next: np.ndarray,
        y_prev: np.ndarray,
        y_now: np.ndarray,
        y_next: np.ndarray,
    ) -> np.ndarray:
        theta1 = np.arctan2(y_now - y_prev, x_now - x_prev)
        theta2 = np.arctan2(y_next - y_now, x_next - x_now)
        return (theta2 - theta1 + np.pi) % (2 * np.pi) - np.pi

    @staticmethod
    def sliding_window_sign_persistence(
        column: pd.Series, window_size: int, beta_persistence: float
    ) -> np.ndarray:
        values = column.fillna(0).to_numpy()
        half_window = window_size // 2
        padded = np.pad(values, (half_window, half_window), mode="reflect")
        shape = (values.size, window_size)
        strides = (padded.strides[0], padded.strides[0])
        windows = np.lib.stride_tricks.as_strided(padded, shape=shape, strides=strides)

        abs_windows = np.abs(windows)
        selected = np.argpartition(-abs_windows, kth=half_window - 1, axis=1)[:, :half_window]
        row_indices = np.arange(windows.shape[0])[:, None]
        signs = np.sign(windows[row_indices, selected])

        positive_counts = np.sum(signs > 0, axis=1)
        negative_counts = np.sum(signs < 0, axis=1)
        threshold = np.ceil(beta_persistence * half_window)
        return (np.maximum(positive_counts, negative_counts) >= threshold).astype(int)
```

hmm_categorical.py

```
"""Categorical hidden Markov model for binary trajectory features.

The emission model is categorical over all 2^D binary feature
combinations. This allows dependencies between the D binary features
within each hidden state, rather than assuming independent Bernoulli
emissions.
"""

from __future__ import annotations

from dataclasses import dataclass
from itertools import product
from typing import Iterable, Sequence

import numpy as np


Array = np.ndarray


@dataclass
class HMMParameters:
    """Parameters of a categorical-emission HMM."""

    transition: Array
    initial: Array
    emissions: Array
    x_values: Array


class CategoricalHMM:
    """Hidden Markov model with categorical emissions.

    Parameters
    ----------
    n_states:
        Number of hidden states.
    n_features:
        Number of binary features in each observation vector.
    transition:
        State-transition matrix A, where A[i, j] = P(Q_{t+1}=j | Q_t=i).
    initial:
        Initial state distribution.
    emissions:
        Emission matrix with shape (n_states, 2**n_features). Each row is a
        probability distribution over the binary feature combinations stored
        in ``x_values``.
    """

    def __init__(
        self,
        n_states: int,
        n_features: int = 3,
        transition: Array | None = None,
        initial: Array | None = None,
        emissions: Array | None = None,
    ) -> None:
        self.n_states = int(n_states)
        self.n_features = int(n_features)
        self.x_values = np.array(list(product([0, 1], repeat=self.n_features)), dtype=int)

        self.transition = self._normalize_rows(
            np.full((self.n_states, self.n_states), 1.0 / self.n_states)
            if transition is None
            else np.asarray(transition, dtype=float)
        )
        self.initial = self._normalize(
            np.full(self.n_states, 1.0 / self.n_states)
            if initial is None
            else np.asarray(initial, dtype=float)
        )
        self.emissions = self._normalize_rows(
            np.full((self.n_states, len(self.x_values)), 1.0 / len(self.x_values))
            if emissions is None
```

```
            else np.asarray(emissions, dtype=float)
        )

        self._validate_shapes()

    @classmethod
    def from_parameters(cls, params: HMMParameters) -> "CategoricalHMM":
        model = cls(
            n_states=params.transition.shape[0],
            n_features=params.x_values.shape[1],
            transition=params.transition,
            initial=params.initial,
            emissions=params.emissions,
        )
        if not np.array_equal(model.x_values, params.x_values):
            raise ValueError("Provided x_values do not match binary feature ordering.")
        return model

    def parameters(self) -> HMMParameters:
        return HMMParameters(
            transition=self.transition.copy(),
            initial=self.initial.copy(),
            emissions=self.emissions.copy(),
            x_values=self.x_values.copy(),
        )

    def fit(
        self,
        observation_sequences: Sequence[Array],
        n_iter: int = 50,
        update_transition: bool = True,
        tol: float | None = None,
    ) -> list[float]:
        """Estimate HMM parameters using the Baum-Welch algorithm.

        Parameters
        ----------
        observation_sequences:
            List of arrays with shape (T_i, n_features).
        n_iter:
            Maximum number of EM iterations.
        update_transition:
            Whether to update the transition matrix during EM.
        tol:
            Optional convergence tolerance on the log-likelihood increase.

        Returns
        -------
        list of float
            Log-likelihood values after each iteration.
        """
        sequences = [self._as_observations(obs) for obs in observation_sequences]
        log_likelihoods: list[float] = []

        for _ in range(n_iter):
            gammas = []
            xis = []
            log_likelihood = 0.0

            for obs in sequences:
                alpha, scales = self._forward(obs)
                beta = self._backward(obs, scales)
                gamma, xi = self._posterior(obs, alpha, beta)
                gammas.append(gamma)
                xis.append(xi)
                log_likelihood += -np.sum(np.log(scales))

            self.initial = self._normalize(np.mean([gamma[0] for gamma in gammas], axis=0))

            if update_transition:
                xi_sum = np.sum([xi.sum(axis=0) for xi in xis], axis=0)
                self.transition = self._normalize_rows(xi_sum)

            self._update_emissions(sequences, gammas)
            self._clip_probabilities()
            log_likelihoods.append(float(log_likelihood))

```

```
            if tol is not None and len(log_likelihoods) > 1:
                if abs(log_likelihoods[-1] - log_likelihoods[-2]) < tol:
                    break

        return log_likelihoods

    def predict_viterbi(self, observations: Array) -> Array:
        """Return the most likely hidden-state sequence using Viterbi."""
        obs = self._as_observations(observations)
        emission_probs = self.emission_probabilities(obs)
        n_obs = len(obs)

        delta = np.zeros((n_obs, self.n_states), dtype=float)
        psi = np.zeros((n_obs, self.n_states), dtype=int)

        delta[0] = self.initial * emission_probs[0]
        delta[0] = self._normalize(delta[0])

        for t in range(1, n_obs):
            for j in range(self.n_states):
                scores = delta[t - 1] * self.transition[:, j]
                psi[t, j] = int(np.argmax(scores))
                delta[t, j] = scores[psi[t, j]] * emission_probs[t, j]
            delta[t] = self._normalize(delta[t])

        states = np.zeros(n_obs, dtype=int)
        states[-1] = int(np.argmax(delta[-1]))
        for t in range(n_obs - 2, -1, -1):
            states[t] = psi[t + 1, states[t + 1]]
        return states

    def predict_posterior(self, observations: Array) -> Array:
        """Return posterior state probabilities P(Q_t | X_1:T)."""
        obs = self._as_observations(observations)
        alpha, scales = self._forward(obs)
        beta = self._backward(obs, scales)
        gamma = alpha * beta
        return self._normalize_rows(gamma)

    def emission_probabilities(self, observations: Array) -> Array:
        """Return P(X_t | Q_t=i) for all times and states."""
        obs = self._as_observations(observations)
        indices = self._observation_indices(obs)
        return self.emissions[:, indices].T

    def _forward(self, obs: Array) -> tuple[Array, Array]:
        n_obs = len(obs)
        emission_probs = self.emission_probabilities(obs)
        alpha = np.zeros((n_obs, self.n_states), dtype=float)
        scales = np.zeros(n_obs, dtype=float)

        alpha[0] = self.initial * emission_probs[0]
        scales[0] = alpha[0].sum()
        alpha[0] /= scales[0]

        for t in range(1, n_obs):
            alpha[t] = (alpha[t - 1] @ self.transition) * emission_probs[t]
            scales[t] = alpha[t].sum()
            alpha[t] /= scales[t]
        return alpha, scales

    def _backward(self, obs: Array, scales: Array) -> Array:
        n_obs = len(obs)
        emission_probs = self.emission_probabilities(obs)
        beta = np.ones((n_obs, self.n_states), dtype=float)

        for t in range(n_obs - 2, -1, -1):
            beta[t] = self.transition @ (emission_probs[t + 1] * beta[t + 1])
            beta[t] /= scales[t + 1]
        return beta

    def _posterior(self, obs: Array, alpha: Array, beta: Array) -> tuple[Array, Array]:
        emission_probs = self.emission_probabilities(obs)
        n_obs = len(obs)
        gamma = self._normalize_rows(alpha * beta)
        xi = np.zeros((n_obs - 1, self.n_states, self.n_states), dtype=float)
```

```
        for t in range(n_obs - 1):
            numerator = (
                alpha[t, :, None]
                * self.transition
                * emission_probs[t + 1, None, :]
                * beta[t + 1, None, :]
            )
            denominator = numerator.sum()
            xi[t] = numerator / denominator
        return gamma, xi

    def _update_emissions(self, sequences: Sequence[Array], gammas: Sequence[Array]) -> None:
        weighted_counts = np.zeros_like(self.emissions)
        for obs, gamma in zip(sequences, gammas):
            indices = self._observation_indices(obs)
            for state in range(self.n_states):
                weighted_counts[state] += np.bincount(
                    indices, weights=gamma[:, state], minlength=len(self.x_values)
                )
        self.emissions = self._normalize_rows(weighted_counts)

    def _observation_indices(self, observations: Array) -> Array:
        obs = self._as_observations(observations)
        indices = np.zeros(len(obs), dtype=int)
        for idx, value in enumerate(self.x_values):
            matches = np.all(obs == value, axis=1)
            indices[matches] = idx
        return indices

    def _as_observations(self, observations: Array) -> Array:
        obs = np.asarray(observations, dtype=int)
        if obs.ndim != 2 or obs.shape[1] != self.n_features:
            raise ValueError(f"Expected observations with shape (T, {self.n_features}).")
        if not np.all((obs == 0) | (obs == 1)):
            raise ValueError("Observations must be binary.")
        return obs

    def _validate_shapes(self) -> None:
        if self.transition.shape != (self.n_states, self.n_states):
            raise ValueError("Invalid transition matrix shape.")
        if self.initial.shape != (self.n_states,):
            raise ValueError("Invalid initial distribution shape.")
        if self.emissions.shape != (self.n_states, len(self.x_values)):
            raise ValueError("Invalid emission matrix shape.")

    def _clip_probabilities(self, eps: float = 1e-30) -> None:
        self.initial = self._normalize(np.clip(self.initial, eps, 1.0))
        self.transition = self._normalize_rows(np.clip(self.transition, eps, 1.0))
        self.emissions = self._normalize_rows(np.clip(self.emissions, eps, 1.0))

    @staticmethod
    def _normalize(x: Array) -> Array:
        total = np.asarray(x, dtype=float).sum()
        if total <= 0:
            raise ValueError("Cannot normalize an array with non-positive sum.")
        return np.asarray(x, dtype=float) / total

    @staticmethod
    def _normalize_rows(x: Array) -> Array:
        arr = np.asarray(x, dtype=float)
        row_sums = arr.sum(axis=1, keepdims=True)
        if np.any(row_sums <= 0):
            raise ValueError("Cannot normalize rows with non-positive sums.")
        return arr / row_sums
```

default_parameters.py

```
"""Default HMM parameters used for flight-phase inference."""

from __future__ import annotations

import numpy as np

from hmm_categorical import HMMParameters

# Observation order: [straightness, positive_vertical_speed, curvature_sign_persistence]
X_VALUES = np.array([[i, j, k] for i in range(2) for j in range(2) for k in range(2)], dtype=in[illegible]

# State order inferred from the emission distributions below:
# 0 = climb, 1 = search, 2 = transition.
STATE_NAMES = ("climb", "search", "transition")

TRANSITION_MATRIX = np.array(
    [
        [0.98961359, 0.00346214, 0.00692427],
        [0.00666667, 0.99000000, 0.00333333],
        [0.00099934, 0.00712054, 0.99188012],
    ],
    dtype=float,
)

INITIAL_DISTRIBUTION = np.array([5.66075578e-02, 9.43392442e-01, 5.88699965e-29], dtype=float)

EMISSION_MATRIX = np.array(
    [
        [
            1.54243294e-11,
            5.07106916e-04,
            8.72065952e-05,
            9.98979845e-01,
            8.52234426e-21,
            1.25568196e-12,
            7.85199401e-13,
            4.25841826e-04,
        ],
        [
            3.25680580e-01,
            1.77503496e-01,
            4.95899060e-01,
            7.21547291e-04,
            6.96932383e-05,
            1.93093305e-05,
            1.06025552e-04,
            2.89805651e-07,
        ],
        [
            6.93534587e-06,
            1.04473011e-08,
            2.91554932e-06,
            1.93493842e-10,
            7.38362033e-01,
            1.40643862e-02,
            2.41340621e-01,
            6.22309818e-03,
        ],
    ],
    dtype=float,
)

STRAIGHTNESS_THRESHOLDS_RAD = {
    "paraglider": 0.20960958,
    "hang_glider": 0.17398039,
    "sailplane": 0.10,
}


def default_parameters() -> HMMParameters:
    """Return the default fitted HMM parameters."""
    return HMMParameters(
        transition=TRANSITION_MATRIX,
        initial=INITIAL_DISTRIBUTION,
```

```
        emissions=EMISSION_MATRIX,
        x_values=X_VALUES,
    )
```

postprocessing.py

```python
"""Post-processing utilities for decoded HMM states."""

from __future__ import annotations

import numpy as np
import pandas as pd


def rolling_majority(series: pd.Series, window: int) -> pd.Series:
    """Smooth a categorical series using a centered rolling majority vote."""
    if window <= 1:
        return series.copy()

    codes, categories = pd.factorize(series, sort=True)
    coded = pd.Series(codes, index=series.index)
    n_categories = len(categories)
    counts = np.empty((len(series), n_categories), dtype=np.int32)

    for category in range(n_categories):
        indicator = (coded.to_numpy() == category).astype(np.int8)
        counts[:, category] = (
            pd.Series(indicator, index=series.index)
            .rolling(window=window, center=True, min_periods=1)
            .sum()
            .to_numpy()
        )

    smoothed = pd.Series(categories[counts.argmax(axis=1)], index=series.index)
    try:
        smoothed = smoothed.astype(series.dtype)
    except (TypeError, ValueError):
        pass
    return smoothed
```

infer_flight_phases.py

```python
"""Infer transition/search/climb phases for a single flight track."""

from __future__ import annotations

import argparse
from pathlib import Path

import pandas as pd

from default_parameters import STATE_NAMES, STRAIGHTNESS_THRESHOLDS_RAD, default_parameters
from hmm_categorical import CategoricalHMM
from postprocessing import rolling_majority
from trajectory_features import FeatureConfig, TrajectoryFeatureFactory


def read_track(path: str | Path) -> pd.DataFrame:
    """Read a track from pickle or CSV."""
    path = Path(path)
    if path.suffix.lower() in {".pkl", ".pickle"}:
        return pd.read_pickle(path)
    if path.suffix.lower() == ".csv":
        return pd.read_csv(path)
    raise ValueError("Input track must be a .pkl, .pickle, or .csv file.")


def infer_regimes(
    track: pd.DataFrame,
    glider_type: str = "paraglider",
    smoothing_window: int = 6,
    persistence_window: int = 30,
    beta_persistence: float = 0.9,
    state_smoothing_window: int = 90,
) -> pd.DataFrame:
    """Return the input track with raw and smoothed HMM regimes."""
    if glider_type not in STRAIGHTNESS_THRESHOLDS_RAD:
        raise ValueError(f"Unknown glider_type: {glider_type}")

    feature_config = FeatureConfig(
        smoothing_window=smoothing_window,
        persistence_window=persistence_window,
        beta_persistence=beta_persistence,
        straightness_threshold_rad=STRAIGHTNESS_THRESHOLDS_RAD[glider_type],
    )
    factory = TrajectoryFeatureFactory(feature_config)
    observations = factory.transform(track)

    model = CategoricalHMM.from_parameters(default_parameters())
    raw_states = model.predict_viterbi(observations)
    raw_regimes = pd.Series([STATE_NAMES[state] for state in raw_states], index=track.index)
    smoothed_regimes = rolling_majority(raw_regimes, state_smoothing_window)

    output = track.copy()
    output["regime_raw"] = raw_regimes
    output["regime"] = smoothed_regimes
    return output


def main() -> None:
    parser = argparse.ArgumentParser(description=__doc__)
    parser.add_argument("input", help="Input track file (.pkl, .pickle, or .csv).")
    parser.add_argument("output", help="Output CSV file.")
    parser.add_argument(
        "--glider-type",
        default="paraglider",
        choices=sorted(STRAIGHTNESS_THRESHOLDS_RAD),
        help="Aircraft type used to choose the straightness threshold.",
    )
    args = parser.parse_args()

    track = read_track(args.input)
    output = infer_regimes(track, glider_type=args.glider_type)
    output.to_csv(args.output, index=False)

```

```
if __name__ == "__main__":
    main()
```

train_hmm.py

```python
"""Train the categorical HMM from preprocessed flight tracks."""

from __future__ import annotations

import argparse
from pathlib import Path

import numpy as np
import pandas as pd

from hmm_categorical import CategoricalHMM
from trajectory_features import FeatureConfig, TrajectoryFeatureFactory


def load_tracks(input_dir: Path) -> list[pd.DataFrame]:
    """Load all pickle and CSV tracks from a directory."""
    paths = sorted(list(input_dir.glob("*.pkl")) + list(input_dir.glob("*.pickle")) + list(inpu
    tracks = []
    for path in paths:
        if path.suffix.lower() in {".pkl", ".pickle"}:
            tracks.append(pd.read_pickle(path))
        else:
            tracks.append(pd.read_csv(path))
    return tracks


def filter_track(track: pd.DataFrame, min_duration_s: int = 3600, max_mean_dt_s: float = 1.2)
    """Return True if a track satisfies basic quality criteria."""
    if not track["time"].is_unique:
        return False
    if len(track) < min_duration_s:
        return False
    mean_dt = track["time"].diff().mean()
    return bool(mean_dt < max_mean_dt_s)


def initial_model() -> CategoricalHMM:
    """Return a physically informed initialization.

    State order: 0 = climb, 1 = search, 2 = transition.
    """
    transition = np.array(
        [
            [0.99, 0.005, 0.005],
            [0.005, 0.99, 0.005],
            [0.005, 0.005, 0.99],
        ]
    )
    initial = np.full(3, 1 / 3)
    emissions = np.array(
        [
            [0.05, 0.14, 0.30, 0.50, 0.0025, 0.0025, 0.0025, 0.0025],
            [0.31, 0.20, 0.20, 0.10, 0.10, 0.03, 0.03, 0.03],
            [0.025, 0.025, 0.025, 0.025, 0.50, 0.10, 0.20, 0.10],
        ]
    )
    return CategoricalHMM(n_states=3, transition=transition, initial=initial, emissions=emissi


def train(input_dir: Path, n_iter: int, straightness_threshold_rad: float) -> CategoricalHMM:
    config = FeatureConfig(straightness_threshold_rad=straightness_threshold_rad)
    factory = TrajectoryFeatureFactory(config)
    observations = []
    for track in load_tracks(input_dir):
        if filter_track(track):
            observations.append(factory.transform(track))

    if not observations:
        raise RuntimeError("No valid tracks were found for training.")

    model = initial_model()
    model.fit(observations, n_iter=n_iter, update_transition=True)
    return model

```

```python

def main() -> None:
    parser = argparse.ArgumentParser(description=__doc__)
    parser.add_argument("input_dir", help="Directory containing track files.")
    parser.add_argument("output_npz", help="Output .npz file for fitted parameters.")
    parser.add_argument("--n-iter", type=int, default=50)
    parser.add_argument("--straightness-threshold-rad", type=float, default=0.20960958)
    args = parser.parse_args()

    model = train(Path(args.input_dir), args.n_iter, args.straightness_threshold_rad)
    params = model.parameters()
    np.savez(
        args.output_npz,
        transition=params.transition,
        initial=params.initial,
        emissions=params.emissions,
        x_values=params.x_values,
    )
    print("Transition matrix:\n", params.transition)
    print("Initial distribution:\n", params.initial)
    print("Emission matrix:\n", params.emissions)


if __name__ == "__main__":
    main()
```

simulation_check.py

```python
"""Small synthetic-data check for the categorical HMM implementation."""

from __future__ import annotations

import numpy as np

from hmm_categorical import CategoricalHMM


def simulate_hmm(
    n_obs: int,
    transition: np.ndarray,
    initial: np.ndarray,
    emissions: np.ndarray,
    rng: np.random.Generator,
) -> tuple[np.ndarray, np.ndarray]:
    n_states, n_symbols = emissions.shape
    x_values = np.array([[i, j, k] for i in range(2) for j in range(2) for k in range(2)])
    states = np.zeros(n_obs, dtype=int)
    symbols = np.zeros(n_obs, dtype=int)

    states[0] = rng.choice(n_states, p=initial)
    symbols[0] = rng.choice(n_symbols, p=emissions[states[0]])
    for t in range(1, n_obs):
        states[t] = rng.choice(n_states, p=transition[states[t - 1]])
        symbols[t] = rng.choice(n_symbols, p=emissions[states[t]])
    return states, x_values[symbols]


def main() -> None:
    rng = np.random.default_rng(42)
    transition = np.array([[0.95, 0.03, 0.02], [0.02, 0.95, 0.03], [0.02, 0.03, 0.95]])
    initial = np.array([0.5, 0.3, 0.2])
    emissions = np.array(
        [
            [0.01, 0.01, 0.01, 0.90, 0.02, 0.02, 0.02, 0.01],
            [0.50, 0.20, 0.20, 0.02, 0.02, 0.02, 0.02, 0.02],
            [0.02, 0.02, 0.02, 0.02, 0.70, 0.08, 0.10, 0.04],
        ]
    )
    emissions = emissions / emissions.sum(axis=1, keepdims=True)
    states, observations = simulate_hmm(3000, transition, initial, emissions, rng)

    model = CategoricalHMM(n_states=3, transition=transition, initial=initial, emissions=emiss[illegible]
    model.fit([observations], n_iter=10)
    predicted = model.predict_viterbi(observations)
    print("Decoded state fractions:", np.bincount(predicted, minlength=3) / len(predicted))
    print("Fitted transition matrix:\n", model.transition)


if __name__ == "__main__":
    main()
```

requirements.txt

```
numpy>=1.23
pandas>=1.5
scipy>=1.9
```
